# G6PD gene variants and its association with malaria in a Sri Lankan population

**DOI:** 10.1186/s12936-015-0603-9

**Published:** 2015-02-22

**Authors:** Rajika L Dewasurendra, Kirk A Rockett, S Deepika Fernando, Richard Carter, Dominic P Kwiatkowski, Nadira D Karunaweera, in collaboration with the MalariaGEN Consortium

**Affiliations:** Department of Parasitology, Faculty of Medicine, University of Colombo, 25, Kynsey Road, Colombo 08, Sri Lanka; TheWellcome Trust Centre for Human Genetics, University of Oxford, Roosevelt Drive, Oxford, OX3, 7BN UK; Division of Biological Sciences, Ashworth Laboratories, University of Edinburgh, West Mains Rd, Edinburgh, EH9 3JT UK; Wellcome Trust Sanger Institute, Cambridge, UK

**Keywords:** Haemolysis, SNPs, X chromosome, Red blood cells, Parasitaemia, Disease-severity

## Abstract

**Background:**

Glucose-6-phosphate dehydrogenase (G6PD) is an enzyme that plays an important role in many cellular functions. Deficiency of this enzyme results from point mutations in the coding region of the G6PD gene. G6PD-deficiency is important in malaria, as certain anti-malarial drugs could induce haemolysis in such patients and mutations in this gene may influence the susceptibility or resistance to the disease. Detailed information on genetic variations in the G6PD gene for Sri Lankan populations is yet to be revealed. This study describes a set of G6PD mutations present in a Sri Lankan population and their association with uncomplicated malaria.

**Methods:**

DNA was extracted from 1,051 individuals. Sixty-eight SNPs in the region of the G6PD gene were genotyped. A database created during the 1992–1993 malaria epidemic for the same individuals was used to assess the associations between the G6PD SNPs and parasite density or disease severity of uncomplicated malaria infections. Linkage disequilibrium for SNPs and haplotype structures were identified.

**Results:**

Seventeen genetic variants were polymorphic in this population. The mutant allele was the major allele in 9 SNPs. Common G6PD variants already described in Asians or South-Asians seemed to be absent or rare in this population. Both the severity of disease in uncomplicated malaria infections and parasitaemia were significantly lower in males infected with *Plasmodium falciparum* carrying the ancestral allele of rs915942 compared to those carrying the mutant allele. The parasite density of males infected with *P. falciparum* was significantly lower also in those who possessed the mutant alleles of rs5986877, rs7879049 and rs7053878. Two haplotype blocks were identified, where the recombination rates were higher in males with no history of malaria when compared to those who have experienced the disease in the past.

**Conclusions:**

This is the most detailed survey of G6PD SNPs in a Sri Lankan population undertaken so far that enabled novel description of single nucleotide polymorphisms within the G6PD gene. A few of these genetic variations identified, demonstrated a tendency to be associated with either disease severity or parasite density in uncomplicated disease in males. Known G6PD gene polymorphisms already described from elsewhere were either absent or rare in the local study population.

## Background

Glucose-6-phosphate dehydrogenase (G6PD) is an important enzyme present in all cells that catalyzes the first step of the hexose-monophosphate pathway producing NADPH. Cells require NADPH for many reactions in biosynthetic pathways including maintenance of the effective redox potential that helps to protect red cell membranes against oxidative stress and injury [1]. Deficiencies in G6PD activity give rise to the most common enzymopathy known affecting over 400 million people worldwide. Of the various clinical manifestations that occur due to G6PD deficiency, the most common include neonatal jaundice and acute haemolytic anaemia [1]. Certain drugs (e.g. primaquine), infections or foods (particularly fava beans) can trigger this phenomenon. The resulting haemolysis is believed to be the result of the inability of the G6PD-deficient red cells to withstand the oxidative damage produced either directly or indirectly by these triggering agents.

The G6PD enzyme has over 400 different biochemical variants as reviewed by Beutler and Vuilliamy [2]. The coding gene is located on the X chromosome and about 186 genetic variants, which can induce functional enzyme deficiency in the host have been described [3]. Many G6PD gene variants are named after locations from where they were first discovered; for example, Canton, Mahidol, Kaiping and Viangchang (which have been identified as the most prevalent in Asian populations [4]) and G6PD Mediterranean, Orissa, Kalyan-Kerala, Coimbra (more common Indian variants [3,5,6]). Mutations in this gene are of practical relevance to malaria-infected individuals for two main reasons; On one hand G6PD-deficiency is believed to be associated with susceptibility or resistance to malarial disease while on the other hand, certain oxidative anti-malarial drugs (i.e. primaquine and sulphur containing drugs) can trigger haemolytic anaemia [7-9], which is of practical concern to all malaria endemic countries including Sri Lanka.

Although a few studies have been conducted in Sri Lanka to describe the correlation between drug- induced haemolysis and the functional G6PD enzyme deficiency status in mid 1960s [10,11], there is a void due to lack of recent published data, and no information exists on either the genetic variants associated with this condition or its association with malaria in this country. This study focuses on G6PD genetic variants that prevail in a selected population in South-Eastern Sri Lanka (residents of a known endemic area for both *Plasmodium vivax* and *Plasmodium falciparum* malaria) and their possible association with susceptibility or protection against uncomplicated malarial disease.

## Methods

### Ethical clearance

Ethical clearance for this study was granted by the Ethics Review Committee, Faculty of Medicine, University of Colombo [12]. Informed consent was obtained for all individuals and where participants were below 18-years old, proxy consent was sought from their parents or guardians.

### Study area

The study was conducted in Southern Sri Lanka in eight adjacent villages in the Kataragama Medical Officer of Health (MOH) division in the district of Moneragala namely Angunnara, Old Buttala Road, Kohombadigana, Karawile, New Buttala Road, Sella Kataragama, Thanamalwila Road and Akkarawissa. These villages are situated in the dry zone of the country. The majority of the population in these villages are engaged in agriculture and farming, except in Sella Kataragama, which is a semi-urban area where more residents engaged in occupations such as small-scale trading. This area experiences annual northeast monsoonal rains which has been closely linked with the seasonal transmission of malaria prior to the drastic decline in malaria case numbers experienced across the country from year 2000 onwards [13,14]. The average mosquito inoculation rates of malaria in the study area during 1992–1993 was recorded as 0.5-1 mosquito bites per person per year [13] with the country recording a 285,227 *P. vivax* and 77,970 *P. falciparum* cases [14].

### Participants for the study

The villages selected for this study were similar to an earlier epidemiological study described elsewhere [13,15]. Clinical information was extracted from archived databases at the Malaria Research Station (MRS) Kataragama in order to create a brief history of malaria for each study site and participant (i.e. number of malaria attacks up to the year 1992–1993, malaria species, parasitaemia and disease severity scores of malaria attacks for each individual). Of more than two thousand individuals listed in the databases, 1,051 were traced and were visited between December 2006 and May 2007 in order to collect fresh blood samples for DNA extraction and to collect more a recent malaria history as part of a study on genomic epidemiology of malaria [15].

### Sample and data collection

Two milliliters of blood was collected by venipuncture from all study participants into EDTA–coated tubes prior to DNA extraction. Each tube was labeled with a unique ID number assigned to each individual. The population consisted of people who have had one or more malaria attacks (with laboratory confirmation) during or prior to 1992–1993 (Group A) and individuals with no history of malaria attacks during their lifetime up to 1992–1993 (Group B). The study population was categorized into these 2 groups based on either the information documented in the MRS database (1986 to 1993) or individual's recall memory on history of laboratory-confirmed malarial disease as of 1992–1993 [16].

### DNA extraction and genotyping

Genomic DNA (gDNA) was extracted from blood samples using Nucleon BACC2 DNA extraction kits [Gen-Probe Life Sciences, Tepnel Research Products and Services, Manchester, UK]. Five nano-grammes of gDNA was whole-genome amplified by primer-extension pre-amplification (PEP) using N15 primers (Sigma, UK) and Biotaq (Bioline, UK) polymerase as previously described by Zhang *et al.* [17]. Sixty-eight single nucleotide polymorphisms (SNPs) located in the G6PD gene and its flanking regions were selected from the many hundreds of SNPs identified in the literature and on public databases based on the given criteria (1) previous associations with malaria, (2) predicted functional consequences with respect to G6PD enzyme activity (3) estimated minor allele frequency and (4) whether they made a viable Sequenom assay design [18]. Three gender-typing markers were also included. Assays were performed using the Sequenom® iPLEX platform according to manufacturer’s instruction using diluted PEP DNA (1:10). Genotype calls were made using the Sequenom® Typer v4.03 software [19].

### Data analysis

Data were analysed using SPSS V 15.0 and Haploview (V4.2). Eleven SNPs with greater than 10% missing calls plus 141 individuals with greater than 20% missing calls were excluded from further analysis. Twenty individuals whose genotyped sex failed or mismatched with the recorded sex were also excluded from the study. After quality control of samples and SNPs, 57 SNPs in 890 individuals were selected for further analysis (Table 1).

**Table 1:** **Single Nucleotide polymorphisms selected for analysis**

	**rs number**	**Chromosome**	**Ancestral: Alternate allele**	**Function**
		**Position**	**(MAF)**	
1	rs766420	153554404	*C*: G (0.491)	Intron variant
2	rs915941	153626649	*C*: A (0.12)	5′ UTR variant
3	rs915942	153626738	G :*A* (0.119)	Splice region variant
4	rs28470352	153753490	T :*A* (0.0)	Intergenic variant
5	rs61042368	153755336	G :*A* (0.001)	Downstream gene variant
6	rs12389569	153757734	G :*A* (0.001)	Downstream gene variant
7	NT_011726.13_4578452	153757978	C:*G* (0.0)	
8	rs188196644	153759426	C :*A* (0.0)	Downstream gene variant
9	NT_011726.13_4580014	153759540	G: *A* (0.0)	
10	rs181015082	153759667	G : *A* (0.0)	3′ UTR variant
11	CM973154 (Bangkok_Noi)	153760261	A: *C* (0.0)	
12	rs77214077	153760429	G: *A* (0.0)	Synonymous variant
13	rs72554665 (Canton)	153760484	C: *A* (0.0)	Missense variant
14	rs2071429	153760508	*G*: A (0.452)	Intron variant
15	CM920290 (Union)	153760605	G: *A* (0.0)	
16	rs2230037	153760654	G: *A* (0.413)	Synonymous variant
17	CM067413	153760883	G: *C* (0.0)	
18	rs2230036	153760953	C: *T* (0.001)	Synonymous variant
19	rs137852342 (Chinese-V)	153761184	G: *A* (0.0)	Missense variant
20	rs76723693 (Betica)	153761240	*A*: G (0.0)	Missense variant
21	rs137852327 (Viangchan)	153761337	C: *T* (0.0)	Missense variant
22	rs183394670 (nt3042)	153761515	C: *T* (0.0)	Intron variant
23	rs73573478	153761564	G: *A* (0.002)	Non coding exon variant
24	rs5986990	153761628	G: *A* (0.0)	Non coding exon variant
25	NT_011726.13_4582217	153761743	T: *C* (0.0)	
26	rs2515905	153762075	G: *A* (0.0)	Intron variant
27	rs137852328 (Mexico city)	153762340	C: *T* (0.0)	Missense variant
28	rs5986875	153762392	G: *A* (0.001)	Non coding exon variant
29	rs137852330 (Vancouver2)	153762605	G: *A* (0.0)	Missense variant
30	rs5030868 (Mediterranean)	153762634	G: *A* (0.0)	Missense variant
31	rs5030872 (Santa Maria)	153762655	*T*: A (0.0)	Missense variant
32	rs137852314 (Mahidol)	153762710	C: *T* (0.0)	Missense variant
33	rs2515904	153762771	G: *C* (0.0)	Intron variant
34	NT_011726.13_4583299	153762825	C: *T* (0.0)	
35	CM970547 (Valladolod)	153763462	G: *A* (0.0)	
36	rs78365220 (Vanua Lava)	153763485	A: *G* (0.0)	Missense variant
37	rs1050829	153763492	T: *C* (0.0)	Missense variant
38	rs137852349 (Namoru)	153764211	A: *G* (0.0)	Missense variant
39	rs1050828	153764217	C :*T* (0.0)	Missense variant
40	CM052878 (Songklangarind)	153764223	A: *T* (0.0)	
41	rs762516	153764663	C: *T* (0.0)	Intron variant
42	rs73641103	153769889	G: *A* (0.0)	Intron variant
43	NT_011726.13_4591512	153771038	T: *C* (0.0)	
44	rs113492957	153773062	C: *T* (0.001)	Intron variant
45	NT_011726.13_4593593	153773119	G: *A* (0.0)	
46	NT_011726.13_4593634	153773160	A: *C* (0.0)	
47	rs145036913	153773526	A: *G* (0.001)	Intron variant
48	NT_011726.13_4594688	153774214	T: *C* (0.0)	
49	CM950495 (Honiara)	153774272	T: *C* (0.0)	
50	rs5986992	153776107	C: *A* (0.001)	5′ UTR variant
51	NT_011726.13_4596966	153776492	G: *T* (0.0)	
52	rs5986997	153827549	C: *T* (0.0)	Intergenic variant
53	rs4898389	153827637	*G*: A (0.449)	Intergenic variant
54	rs5986877	153828269	*G*: C (0.452)	Intergenic variant
55	rs7879049	153829693	*G*: A (0.404)	Upstream gene variant
56	rs7053878	153834100	*A*: T (0.113)	Upstream gene variant
57	rs60030796	153836171	A: *G* (0.0)	Downstream gene variant

Fifty seven SNPs selected for assay are listed. These include SNPs in the G6PD gene, SNPs in the immediate downstream of the G6PD gene and SNPs known to have long range LD with the G6PD gene: rs numbers/ or primary IDs assigned for each SNP is indicated (GRch37, Ensembl release 68). Chromosome position, minor allele frequency (MAF) and the function of the polymorphic markers are also stated**.** Minor allele of each SNP is italicized.

As described earlier, the population was categorized into two groups based on the information retrieved from the MRS databases and each individual’s history based on recall memory; individuals who had one or more malaria attacks during 1992 and 1993 (Group A) and individuals with no history of malaria attacks during their lifetime up to1992-1993 (Group B). Age distributions of the 2 groups were comparable.

In addition to the number of malaria infection episodes over 19 months spanning 1992 and 1993, the MRS database also contained information on each malaria episode experienced by study subjects during this period that included the degree of disease severity, causative parasite species, parasite density. The severity of clinical disease in each malaria patient was measured using a previously validated scoring system which assessed 11 symptoms recorded or reported by the patient in integer units on a scale of 0 – 2 or 0 – 3 for each symptom [20]. This allows a maximum clinical score (CLS) of 33 for each individual. Individuals were categorized into three groups based on the clinical scores of the first infection acquired during 1992–1993; Low CLS 0–9, Medium CLS 10–19, and High CLS ≥20. Subjects were also divided into 2 categories (high and low parasitaemic groups) using an arbitrary cut off for parasite density (set at 1 parasite per 1000 red blood cells [~0.1%]). Gender stratified analysis was carried out to compare the parasite density, clinical scores of the groups and their possible association with identified genetic markers.

## Results

### Characteristics of the population

During the latest study period (2006–2007), the ages of the study subjects ranged between 14 to 89 years (mean 22.3 years, median 21 years). This equates to an age range of <12 months-old to 74 years-old (mean 22.3 years-old, median 21 years) at the time of the original malaria survey in 1992–1993. All the subjects were apparently healthy and blood smear-negative for malaria at the point of blood sample collection in 2006–2007. All the subjects belonged to the “Sinhala” race and 51.1% of the individuals were males (n = 426).

### Identification of G6PD gene variants present in the population

The information on genetic variants studied in this population study are summarized in Table 1. Out of the 57 genetic markers that passed quality control (see Methods), 17 SNPs were polymorphic in the Sri Lankan population (Table 1). Eight SNPs were found at a frequency of less than 1% and were excluded from further analysis due to group sizes. The remaining nine SNPs had a minor allele frequency of greater than 10% (5 had the alternate allele as the major allele i.e. rs766420, rs915941, rs2071429, rs4898389 and rs7879049 while the other four SNPs had the ancestral allele as the major allele i.e. rs915942, rs2230037, rs5986877 and rs7053878). Of these nine markers, two were intronic variants, two were intergenic variants and two were upstream variants; and the other three SNPs were 5′ UTR, splice region and non-synonymous variants (Table 1). A number of the SNPs genotyped that have been reported in other populations (*G6PD-Mahidol, G6PD-Mediterranean, G6PD-Canton, G6PD-Viangchang, G6PD-Chinese1&5, G6PD-Valladolid, G6PD-Vancouver2, G6PD-Mexico City, G6PD-Betica and G6PD-Union*) were not polymorphic in this Sri Lankan population (Table 1). Therefore, the analysis was focused on the nine SNPs with a minor allele frequency of greater than 10%. Two of these SNPs are located in the G6PD gene and the other seven in adjacent areas on that chromosome; i.e. four in immediate downstream of G6PD gene and three which are known to be in long range LD with G6PD gene [21].

### Relationships of genetic variants with gender and disease status

The number of females who presented with one or more malaria attacks during 1992 and 1993 (n = 174) was comparable to the number of males with a similar history of malaria attacks (n = 169) in this population (Chi^2^ = 0.998, p = 0.524). The percentage of individuals carrying the alternate alleles in group A (malaria-susceptible) and B (who were apparently protected from uncomplicated malaria disease) were compared using the chi-squared test. The percentage of males carrying the alternate allele for rs2071429 (group A: B frequencies = 0.527:0.420, Chi^2^ = 4.643, p = 0.0312) for rs2230037 (group A: B frequencies = 0.479:0.377, Chi^2^ = 4.349, p = 0.037) and for rs4898389 (group A: B frequencies = 0.338:0.661, Chi^2^ = 4.581, p = 0.031) were significantly different in group A when compared to group B but the percentages between groups A and B were not different when females were considered for any of the markers (Table 2). Of the 343 individuals in group A, 44% had *P. vivax* infections, 31.3% had *P. falciparum* infections and 24.7% have had mixed infections with both *P. vivax* and *P. falciparum* during 1992 and 1993. Chi-squared test was used to compare the number of males and females in high, medium or low CLS groups and high or low parasite density groups with the presence of the alternate allele in group A. Number of females carrying either ancestral or alternate allele of any of the tested markers did not significantly differ in CLS groups or parasite density groups for *P. vivax* or *P. falciparum*. However, in males the alternate allele of rs915942 was significantly higher in the low parasitaemia group and low CLS group (for *P. falciparum*) when compared to the other groups (for CLS: Chi = 15.1, p = 0.019; for parasite density: Chi = 101.1, p = 0.018). But this scenario was not observed in males with *P. vivax* malaria infections.

**Table 2:** **Association of the polymorphic SNPs and the malaria infection status**

	**SNP**	**Associated Allele**	**Group A: Group B frequencies**	**Chi** ^**2**^	**p**
Males (n = 426; Group A = 169)	rs766420	G	0.509:0.486	0.206	0.649
	rs915941	C	0.130:0.109	0.444	0.505
	rs915942	G	0.893:0.891	0.006	0.936
	rs2071429	G	0.527:0.420	4.643	0.031
	rs2230037	A	0.479:0.377	4.349	0.037
	rs4898389	G	0.503:0.414	2.991	0.031
	rs5986877	G	0.500:0.415	2.747	0.097
	rs7879049	G	0.436:0.379	1.269	0.260
	rs7053878	T	0.892:0.862	0.790	0.374
Females (n = 464; Group A = 174)	rs766420	G	0.537:0.528	0.083	0.772
	rs915941	A	0.865:0.852	0.310	0.578
	rs915942	G	0.868:0.853	0.370	0.542
	rs2071429	A	0.563:0.557	0.035	0.851
	rs2230037	A	0.405:0.403	0.003	0.958
	rs4898389	A	0.556:0.528	0.607	0.436
	rs5986877	C	0.549:0.524	0.514	0.473
	rs7879049	A	0.608:0.576	0.850	0.356
	rs7053878	T	0.883:0.841	2.847	0.091

Comparison of the genotypes of the 9 polymorphic SNPs with MAF > 10%; frequencies of the associated allele of (Group A)/ (Group B) individuals are also mentioned. Males and females were compared separately using chi squared test.

Furthermore, the number of males in low parasitaemia group carrying the alternate allele of rs5986877 (Chi = 6.237, p = 0.044), rs7879049 (Chi = 7.292, p = 0.026), and rs7053878 (Chi = 7.591, p = 0.022) who were infected with *P. falciparum* were significantly low when compared to other groups. But the number of males carrying the alternate allele of SNP rs4898389 was significantly high in low parasitaemic group for *P. falciparum* when compared to the other groups (Chi = 6.811, p = 0.033).

### Identification of markers in linkage

**Figure 1:**
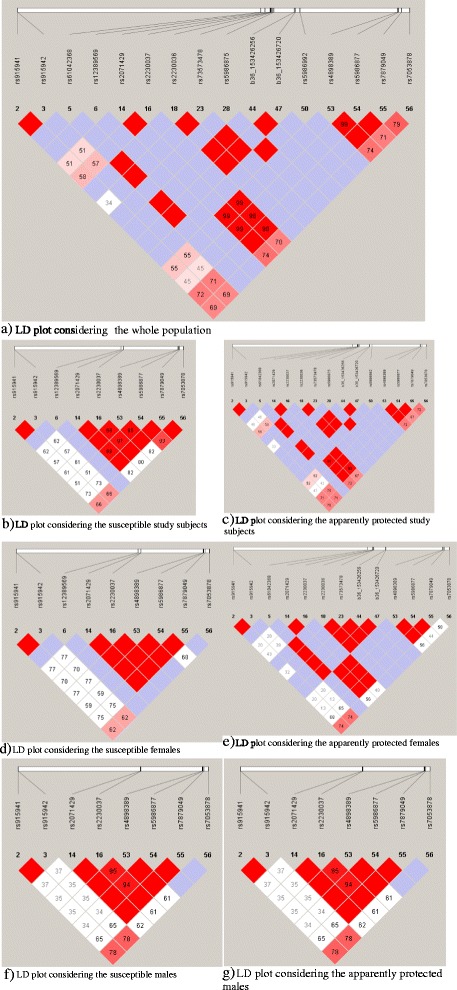
**Linkage disequilibrium (LD) plot of the markers (1a: For the whole population; 1b-1g: for susceptible/protected individuals and gender stratified susceptible and protected population).** Numbers in each box represent 100xD’value. Red squares indicate pairs of SNPs in high linkage (D ~ 1.00). SNPs with minor allele frequency ≥ 0.001 were selected to generate the LD plot and were generated using Haploview (V4.2) software.

**Figure 2:**
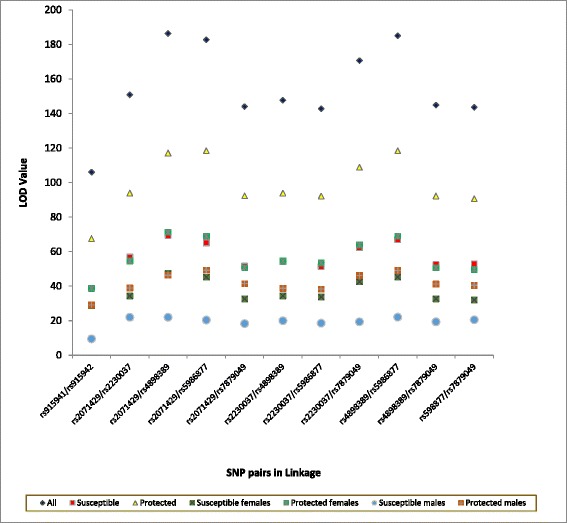
**LOD values of the SNPs in linkage.** SNP pairs which showed D’ ≥ 0.8 and r^2^ ≥ 0.8 are plotted against the LOD value.

Genetic polymorphisms in linkage disequilibrium (LD) were identified using the software Haploview for all individuals. High (D’ ~ 1.00) LD could be observed between several pairs of SNPs identified as polymorphic (Figure 1a-g). Females and males in group B had high LD between these polymorphic SNPs when compared to the females and males in group A (Figure 2). The lowest LOD values (Log of the odds – when 2 loci are in LD) for the SNPs in LD were seen among males in group A. LD was also observed between other SNPs with MAF < 10%, however the low LOD values and r^2^ values may indicate that the LD observed between these markers might be due to chance.

### Identification of haplotypes in the population

Identification of haplotypes was carried out using all 57 tested SNPs in this population. Haplotype blocks were defined using the algorithm described by Wang *et al.* in 2002 [22] and haplotypes were estimated using an accelerated Expectation Maximization (EM) algorithm described by Qin *et al.* in 2002 [23] using Haploview (V 4.2). The blocks were determined for the entire population, for group A and group B separately (Table 3), and for the gender-stratified groups A and B (Table 3). Two haplotype blocks could be identified in all groups except in males in group A. The first haplotype block consisted of two SNPs, i.e. rs915941 and rs915942 (89 bp apart), forming a major haplotype (AG > 87.0%) in all groups (except males in group A). The second haplotype block consisted of 5 SNPs i.e. rs2071429, rs2230037 (SNPs in the G6PD gene), rs4898389, rs5986877 and rs7879049 (SNPs in other locations), with 2 major haplotypes (present in > 35% of the population in all groups) and a minor haplotype (Table 3). The recombination rates between the two haplotype blocks ranged between 0.48 – 0.76, the lowest among the females with apparent protection and the highest among the males with apparent protection from clinical malaria. The susceptible males demonstrated one haplotype block with two major haplotypes each occurring in >40% of individuals in that group and three minor haplotypes (Table 3).

**Table 3:** **Haplotype frequencies**

**Block 1**	**SNPs**		**Frequency of the haplotype**									
	**rs915941**	**rs915942**	**population**	**Group A**	**Group B**	**Males**	**Females**	**Males**	**Group B**			
						**Group A**	**Group A**	**Group B**	**Females**			
Haplotypes	A	G	0.880	0.890	0.870	-	0.870	0.878	0.871			
	*C*	*A*	0.119	0.110	0.124	-	0.120	0.122	0.126			
**Block 2**	**SNPs**					**Frequency of the haplotype**						
	**rs2071429**	**rs2230037**	**rs4898389**	**rs5986877**	**rs7879049**	**Population (0.64)**	**Group A (0.73)**	**Group B (0.77)**	**Males Group A (N/R)**	**Females Group A (0.77)**	**Males Group B (0.76)**	**Females Group B (0.48)**
Haplotypes	A	G	A	C	A	0.545	0.553	0.540	0.475	0.540	0.585	0.516
	*G*	*A*	*G*	*G*	*G*	0.402	0.394	0.407	0.475	0.365	0.364	0.431
	*G*	G	*G*	*G*	A	0.034	0.033	0.034	0.013	0.041	0.028	0.038

Population frequencies of the G6PD haplotypes (>1%) are presented with the minor alleles of each SNP is italicized. Haplotype analysis was conducted for the five markers with the alternate allele, in which four were in linkage disequilibrium. The identified haplotype blocks for the entire population, Group A, Group B, Males/ Females in Group A and B are tabulated below. Recombination rates of the two haplotype blocks are indicated within brackets (N/R = Not Relevant).

## Discussion

This study was conducted to identify variants within the G6PD gene present in a population residing in a malaria-endemic area in southern Sri Lanka. Although there had been a few studies described in the past in Sri Lanka on G6PD enzyme functional deficiency [10,11], there are no studies, as far as it is known to identify genetic polymorphisms and nucleotide variability of the G6PD gene and no information exists on its association with malaria. The 57 SNPs that were studied in the G6PD gene are known coding variants and/or previously identified through association studies with G6PD deficiency *per se* or with malaria, and all selected markers made valid Sequenom assays.

Although some G6PD gene variants including G6PD *Mahidol* variant (rs137852314), *Mediterranean* variant (rs5630868), Canton variant (rs72554665) and *Viangchan* variant (rs13785237), G6PD *Kaiping* (rs72554664) and G6PD *Vanua Lava* (rs78365220) have been established as those that prevail in Asian populations, none of these SNPs were found to be polymorphic in the Sri Lankan population studied. Similar findings with regard to the *Mediterranean* variant has been reported previously on a limited number of samples tested from a different location within Sri Lanka [24].

Of the nine markers with MAF > 10%, 4 SNPs i.e. rs915942, rs2230037, rs5986877 and rs7053878 the alternate allele was the minor allele. These studies are in line with data available online on Asian populations [25]. However, it is interesting to note that the allele frequencies of the alternate alleles of rs2230037 and rs5986877 are much higher when compared to a general Asian population as reported previously (Table 4). Moreover, the minor allele for rs766420 in the Sri Lankan population was the ancestral allele (C) whereas the minor allele in the general Asian population was the alternate allele (G). Furthermore, the minor allele frequencies of rs2071429, rs4898389 and rs7879049 (for which the minor allele is the ancestral allele) are higher when compared to the minor allele frequencies of these markers in the general Asian population.

**Table 4:** **Frequencies of the alternate alleles of the nine major markers present in the study population**

**SNP**	**Study population (Sri Lanka)**	**Asia**	**Africa**
**rs766420 (G)**	51%	23%	61%
**rs915941 (A)**	88%	90%	48%
**rs915942 (A)**	12%	10%	39%
**rs2071429 (A)**	55%	77%	12%
**rs2230037 (A)**	41%	07%	26%
**rs4898389 (A)**	55%	90%	12%
**rs7879049 (A)**	60%	93%	68%
**rs5986877 (C)**	55%	10%	12%
**rs7053878 (T)**	89%	86%	94%

The frequency of the alternate allele in the study population was compared with the given frequencies of the alternate allele of two continents [25]. The alternate allele is indicated within brackets.

These differences might be due to the fact that the genetic variants of Han-Chinese or Japanese population (which are different from the South-Asian or Sri Lankan populations) may have contributed largely to the frequencies calculated for the general Asian populations [25]. The variants G6PD *Punjab*, *Kalyan-Kerala* although identified as common in neighbouring Indian populations were not typed as they could not be designed as SQNM assays.

Of the 57 SNPs genotyped, nine (of which two are in the G6PD gene) had frequencies greater than 10% in this Sri Lankan population. The observations made regarding the males who carried the alternate alleles for rs2071429 and rs2230037 being more susceptible for malaria (Table 2) suggest a gender-biased effect for the mutant type of these SNPs. However homozygous or heterozygous females with the alternate allele of these SNPs did not show any association with malaria disease status.

Population studies on relationships between genetic polymorphisms in the G6PD gene and phenotypic characteristics in uncomplicated malaria are rare. Louicharoen *et al.* [9] observed that the G6PD *Mahidol* variant (found in a Thai population) was associated with a significant reduction in *P. vivax* parasite density both in heterozygous and homozygous females as well as in hemizygous males, whereas no association was observed with *P. falciparum* parasite density. This study however, looked at the G6PD *Mahidol* variant only, whereas the current study analyzed several G6PD variants. The degree of association (protection/susceptibility) with the disease status might differ according to the type of mutation and of course from population to population. A similar case–control study done in Afghanistan by Leslie *et al.* [26] has also shown that the association between G6PD gene mutations and protection against vivax malaria is seen less often in heterozygous females when compared to hemizygous males. The current study confirms the observations made by Leslie *et al*. [26] that either protection and/or susceptibility to the disease are enhanced by mutations in G6PD gene in hemizygous males rather than in heterozygous females, indicating that mono-allelic status of X-linked markers might have a correlation with the disease status rather than the bi-allelic status of the markers in females. Furthermore, the results of the present study indicate that it is not enhanced in homozygous females either. Random inactivation of one of the two inherited X chromosomes does occur in females. Therefore, although genotyped as homozygote with the ancestral or the alternate allele the actual proportion of females that express the gene activity might be much lower, therefore, the relationship between the marker and the disease status may not be evident.

Bienzle *et al.* [27] had reported lower parasite densities (as an indication of disease severity) in two groups of G6PD non-deficient male individuals with different variants of G6PD enzyme activity. The current study reveals male cases carrying the ancestral allele of rs915942 had significantly lower parasite densities for *P. falciparum* when compared to males carrying the alternate allele. However, since G6PD enzyme functional assays were not carried out, it is not possible to comment on any possible associations with the functional status of this enzyme in the study subjects.

Reports on comparisons between G6PD gene mutations and disease status of severe and complicated malaria are abundant. A large-scale study done in Kenya by Ruwende *et al.* [7] revealed that mutations in G6PD are associated with reduction in the risk of severe malaria for both hemizygous males and heterozygous females. However, in two studies done in Mali and Pakistan there was no such association between heterozygous females and severe malaria which is believed to be due to the control group being individuals with ‘uncomplicated malaria’ compared to the cases of severe and complicated malaria [8,26,28]. In the current study, the study subjects who had one or more malaria infections during the study period in 1992–1993 (group A) were compared against the individuals (group B) who at the point of data collection (1992–1993) have never acquired malaria during the lifetime according to the records maintained in the field site and subjects’ history based on his or her memory. Therefore, there may have been inaccuracies in the categorization of “protected individuals” and “susceptible individuals” that may have reduced the power of evidence towards the relationship between the mutant genetic markers and malaria. Guindo *et al.* [8] observed that male individuals with mutations in G6PD (G6PD A- mutation at nucleotide position 202) were protected from severe malaria. This protection was found only in males but not in heterozygous females. More recently, a large multi-centre cases-control study of severe malaria involving 12,000 cases and 17,000 controls demonstrated a highly significant association of G6PD-202 with risk of severe malarial anaemia while a small but significant protection with cerebral malaria [19]. This not only supports the notion that balancing selection occurs in G6PD, but that the relationship of G6PD with disease status is very complex. Moreover a recently study of genetic structure with enzyme function showed a possible interaction between G6PD polymorphisms modifying the outcome [18].

It is, therefore, important to note that there is a major impact of natural selection that occur in malaria hyper-endemic areas, such as Africa, where the numbers of G6PD-deficient homozygous females in populations are very rare [8]. However, this is unlikely to have happened in this study population considering the low and seasonal malaria transmission that prevailed in Sri Lanka, from as far back as it is known. This very fact and the genetic variations between the African and Asian populations may account for the differences observed in this study.

Very high linkage disequilibrium was seen between seven of the nine SNPs with MAF > 10%. Complete linkage disequilibrium could be observed between rs915941 and rs915942, and a similar scenario has been observed in other populations as well (e.g. Gujarati Indians and Han Chinese populations). This could be perhaps due to the very close chromosome locations; 153626649, 153626738 respectively [25]. Unusually high levels of linkage disequilibrium among the markers within G6PD gene has also been observed by Saunders *et al.* [29]. Furthermore, as many of these tested SNPs are in linkage disequilibrium it could be assumed that there might be a combined effect of these host genetic markers towards malaria disease status. When multiple markers in linkage disequilibrium are studied to assess their association with disease traits, haplotype analysis may be more efficient and accurate than separate analyses of the genetic markers. With this premise and with the finding that rs925941 and rs915942 (Block 1) and rs2071429, rs2230037, rs4898389, rs5986877 and rs7879049 (Block 2) fall into the same haplotype blocks are likely to indicate a possible collective effect of these SNPs over malaria infection.

## Conclusions

Single Nucleotide Polymorphisms within the G6PD gene are described in this study population from Sri Lanka. This is the first time that such a dense set of G6PD polymorphisms have been investigated and identified in a Sri Lankan population. Furthermore, it was evident that the common mutations previously described in the Asian or South Asian region are absent or rare in the local population. In light of this further studies to investigate the full spectrum of mutations in Sri Lanka population would help not only understand more on the ancestry of this gene in Sri Lanka but also aid the functional significance of these observed polymorphisms. The study also provides evidence for likely associations of these variations either individually and/or collectively with traits of uncomplicated malaria. Further investigations are underway to combine detailed genetic analyses with functional enzyme studies, which are likely to reveal more information.
